# A community-based intervention to increase participation in cervical cancer screening among immigrants in Norway

**DOI:** 10.1186/s12874-019-0795-8

**Published:** 2019-07-12

**Authors:** Samera A. Qureshi, Abdi Gele, Prabhjot Kour, Kathy A. Møen, Bernadette Kumar, Esperanza Diaz

**Affiliations:** 1 Norwegian Centre for Migrant & Minority Health (NAKMI), Norwegian Institute of Public Health (NIPH), P.O.Box 222 Skøyen, 0213 Oslo, Norway; 2 Norwegian National Advisory Unit on Concurrent Substance Abuse and Mental Health Disorders, Innlandet Hospital Trust, P.O.Box 104, 2381 Brumunddal, Norway; 3 Department of Global Public Health and Primary Care, University of Bergen, P.O.Box 7804, N-5020 Bergen, Norway

**Keywords:** Immigrants, Community-based, Cervical Cancer, Screening, Methods

## Abstract

**Background:**

The attendance to cervical cancer screening is low among immigrants in many high-income countries. Although several interventions have been experimentally tested,implementation remains a challenge. Several factors are an impediment, including the lack of methodological descriptions of the development and implementation of such interventions. In this paper,we present in detail the development, methodological challenges and practical implementation of a community based intervention aimed to increase the participation of immigrant women in cervical cancer screening in Norway.

**Methods:**

This study was initially designed as a cluster randomized trial to be carried out in four geographical areas near Oslo between Feb-October 2017. Participants were immigrant women aged 25–69 years from Pakistan and Somalia. This paper describes the theoretical background for the development of the intervention,followed by challenges,the changes in the original design and solutions adopted related to the study design,recruitment and implementation of the intervention. The intervention was developed based on two theoretical frameworks, the Ecological and the Heron’s six categories intervention framework. An oral 20–25 min presentation in the language of participants encompassing topics of cervical cancer and screening was given according to the needs detected in focus groups conducted at the beginning of the study,followed by an opportunity to raise questions and answering a short questionnaire.

**Results:**

Contrary to the initial study design, this had to be converted into a non-randomised trial due to the difficulties associated with randomization of immigrant families who are finely scattered in heavily populated towns and a high risk of contamination. We therefore adopted a pragmatic approach and recruited women in the intervention areas through a variety of channels and institutions. Neighboring areas were considered to be non-randomised controls. Female researchers with Pakistani and Somali background invited as many women as possible in the intervention areas. Among the women who were invited to participate,42% of the Pakistani and 78% of Somali attended the meetings.

**Conclusion:**

Despite the careful development of a culturally adapted health intervention in collaboration with the community; randomization and recruitment of immigrants for community trials remains challenging. Nevertheless, sharing strategies to overcome specific challenges related to promoting health interventions for immigrants, can be of potential help to scale-up interventions and for building new research projects.

**Trial registration:**

NCT03155581. Retrospectively registered, on 16 May 2017.

**Electronic supplementary material:**

The online version of this article (10.1186/s12874-019-0795-8) contains supplementary material, which is available to authorized users.

## Background

Cervical cancer is the second most commonly diagnosed cancer and third leading cause of cancer deaths among females worldwide [1]. A substantial number of cervical cancer cases and deaths can be prevented if screened and detected early. Screening programs in high-income countries have reduced cervical cancer rates up to 65% over the past 40 years [2]. This is in contrast to countries like Somalia where the rates continue to remain high (34.8/100,000) [1]. This high prevalence of cervical cancer in middle- and low-income countries is largely attributed to either the absence of an organized screening program or a low uptake of screening tests [3, 4].

Immigrant women residing in high-income countries have lower participation in screening tests as compared to the general population [5] . Similarly, in Norway screening uptake among the majority population is higher than among immigrant women (Immigrants were defined based on the definition given by the SSB *“Immigrants are defined as those born outside of Norway to one or two foreign-born parents and four foreign-born grandparents”*) [6]. Women, especially from Somalia, who are among the largest non-Western immigrant groups in Norway, have specifically been observed to have low attendance rates [7]. Though several intervention studies have been conducted to increase the cervical cancer screening attendance among immigrant women in high-income countries [8–11], the results are modest as participation of immigrant women still remains generally low.

Our recent study [12] among Pakistani and Somali immigrant women in Norway, documented individual, sociocultural and health system-related barriers that prevent these women from undergoing screening tests. In parallel, health care professionals interviewed in order to obtain their views regarding the reasons for low attendance to the cervical cancer screening program contributed with knowledge about specific barriers with immigrants [6]. Building upon the opinions and wishes of both groups, we developed an intervention specifically targeting women from Pakistan and Somalia. Although we intended to learn from previous intervention studies with similar aims, our literature review showed that previous intervention studies often lack a detailed description of the design, reliable elements of the intervention itself and its implementation [13, 14]. Also in other health care interventions targeting immigrants, authors seldom provide detailed descriptions about the process of development and implementation of interventions, what functioned well and what went wrong [15]. We tried In order to fill this gap and to aid the advancement of future studies, this paper describes both the development and implementation of a randomised community-based intervention among immigrant women of Pakistani and Somali background living in Norway, along with the process, challenges faced and strategies used to overcome them.

## Methods

Although the CONSORT guidelines for clinical trials (http://www.consort-statement.org/) is not applicable to our manuscript as described in detail below, but we have tried to adhere to the checklist as much as possible.

### Study design and randomization

This intervention study was originally designed as cluster randomised intervention study, and geographic areas were randomized as clusters. Selection of the geographical areas was based on the number of immigrant women residing there. Sample size was calculated for an increase in cancer screening participation from 0.45 to 0.55 with 80% power and 5% significance level. We tried several intra class correlation (ICC) levels, and ended-up dividing the area in 16 clusters that were matched according to the calculated number of female immigrants aged 20 to 69 from Somalia and Pakistan. In our study, all women from Somalia and Pakistan living in the intervention clusters were meant to comprise the intervention groups; immigrant women from the same countries of origin living in control clusters were to be control participants. An estimated 625 and 915 women [16] from Somalia and Pakistan respectively lived in the study area (Akershus and Buskerud). Approximately half of the women from each county were to be in the intervention clusters. After assuring that the cluster-areas were not naturally linked by a mosque or other natural known gathering centre for these populations by looking in a map, clusters were randomly assigned to intervention or control groups.

In the following we will describe 1) the intervention and its elements, 2) the challenges and solutions related to the study design, and recruitment of participants and 3) a description of the actual participants in the intervention based on a short questionnaire applied at the end of the intervention. Information from this questionnaire was entered into excel sheet, and transferred to Stata for analysis. All procedures adopted ensured confidentiality and were according to approval of the ethical committee. The participants gave verbal consent for participation and written consent was not deemed necessary as we did not collect any sensitive information (name, address etc.) and no invasive procedures were conducted.

## Results

### The intervention and its elements

**Fig. 1:**
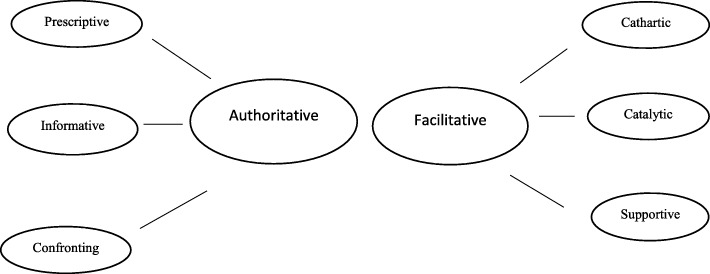
The Heron’s six category intervention framework. Authoritative and facilitative categories by Heron

This community-based intervention was developed based on the Ecological framework identified in our previous study [12, 17] and the Heron’s six category intervention framework (Fig. 1) [18]. This conceptual framework was developed by John Heron [19–21] and classifies interventions in six categories: prescriptive (advice), informative, confronting (challenging), cathartic (enabling the expression of pent-up emotions), catalytic (‘drawing out’) and supportive (confirming or encouraging). Heron’s model is considered as being conceptual for understanding interpersonal relationships, as well as an assessment tool for identifying a range of possible therapeutic interactions between two people [22]. The main purpose of using the Heron’s model here is that we wanted the women to be interactive during the intervention in order to challenge the information being given to them. This would further allow us to address their confusions and thus help and motivate them to participate in screening. Based on the presented theoretical concepts, the factors identified by focus group discussions (6, 12) and in consultation with the research group, we developed an intervention consisting of an informative power-point presentation of 20–25 min (Additional file 1). The presentation content started from a more general description of our project aims followed by brief information on healthy lifestyle and preventative health care and then narrowing it down to cervical cancer. In very simple language (Somalia or Urdu), we explained what do we mean by cervical cancer, its anatomical location -through diagrammatic illustration-, causes, risk factors, and development, This was followed by practical information on cervical cancer screening including the procedure itself and the instruments used, which were also shown physically. A short video clip of the test being done was played. After the video clip the women were informed about the Norwegian screening guidelines for cervical cancer. Towards the end the women were given information on how to book an appointment with their GP’s for taking a screening test, including the payment. The content of the presentation (power point) was both in English and in the respective languages (Urdu and Somali). At the end of the meetings, we distributed a simple questionnaire (Additional file 2) among the participants to obtain demographic data and information regarding previous attendance to cervical cancer screening and knowledge about the health system presented in Table 1. During the refreshment session after the intervention, the researchers intermingled with the participants to chat about whether the information given to them was helpful in their understanding of the problem and if they would actually go for the screening after attending this session.

**Table 1:** Characteristics of the study participants. Characteristics of Pakistani and Somali women who attended the meetings

Variables	Pakistani *n* = 102(%)	Somali *n* = 128(%)
Age (years)		
< 25	1 (0.98)	12 (9.3)
25–45	67 (66.7)	97 (75.7)
46–69	31 (30.4)	17 (13.2)
70+	3 (2.94)	1 (0.8)
Number of Children		
0	5 (4.9)	29 (22.6)
1	8 (6.9)	15 (2.6)
2	20 (18.7)	19 (14.8)
3+	69 (67.7)	62 (48.4)
Marital status		
Single	6 (5.7)	15 (2.6)
Married	87 (86.2)	59 (46.1)
Separated/Divorced/Widow	9 (7.9)	45 (35.1)
Country of birth		
Pakistan	76 (75.6)	–
Somalia	–	123 (96.1)
Norway/other	26 (25.6)	4 (3.1)
Language		
Urdu/Somali	34/− (32.9)	−/112 (87.5)
Norwegian	5 (4.9)	–
Norwegian/Urdu/Somali	43 (41.9)	10 (7.8)
Norwegian/Urdu/Punjabi/English	20 (19.7)	2 (1.5)
Years of school^a^		
No school	12 (11.7)	21 (16.4)
≤ 11	14 (13.6)	41 (32.3)
12–14	38 (37.6)	51 (39.2)
15+	35 (34.7)	6 (4.6)
P for trend		
Do you know how to make an appointment with your GP?		
Yes	90 (89.6)	103 (80.4)
No	12 (11.9)	21 (16.4)
Have you ever taken a screening test?		
Yes	73 (72.7)	58 (45.3)
No	29 (28.4)	68 (51.7)
How long ago		
Never	29 (28.4)	68 (51.7)
1 year	26 (25.7)	19 (14.8)
2+ years	47 (46.0)	42 (32.7)
After this meeting, do you think you should contact your general practitioner to take a screening test?		
Yes	79 (77.4)	116 (90.6)
No	23 (21.9)	12 (9.3)
How often do you need to have someone help you when you read instructions, pamphlets, or other written material from your doctor or pharmacy?		
Never	60 (59.2)	72 (56.2)
Always	12 (11.9)	19 (14.9)
Sometimes	13 (12.1)	21 (16.4)
Rarely	16 (15.8)	15 (11.8)

^a^No school(no education); ≤ 11 years (Elementary school); 12–14 years (High School);15+ (University Level)

The intervention was conducted separately for the Somali and Pakistani communities, in venues (community centre) located in each of the setting areas, depending on the availability and the convenience for the participants. The intervention was conducted by the principal author for the Pakistani group, in collaboration with another female assistant. For the Somali group, a female fieldworker recruited the participants and was present at the meetings, but the intervention was led by a male Somali nurse who had previously been involved in other intervention projects among the Somali women and who is popular within the community.

### Challenges and solutions related to the study design and recruitment

#### Study design

As explained in the methods, our intervention study was originally designed as a community-based cluster randomised intervention study. However, as both groups of immigrants meet each other relatively often due to family or group reunions. Further, when we started the recruitment, we realized that it was difficult to locate immigrant households as they are finely dispersed across the study areas. In addition to that keeping the intervention and control group separate to avoid contamination was also a challenge, because frequent interaction among immigrants’ is much more existent as compared to the majority population. Therefore, after long discussion with researchers and research assistants, it was decided to drop randomization as planned, and adopt direct recruitment of women from different community institutions, households, and religious sites in the every area as a whole. The adjacent areas were then redefined as non-randomised controls.

#### Recruitment

The intervention was carried out from Feb – October 2017. Although we intended to finish by May, but the recruitment was hindered first by the Easter holidays (it is quite a common practice in Norway that immigrants travel to their home countries during the Easter vacations as the schools are closed for almost 2 weeks), during which a lot of women were travelling. Secondly, the holy month of Ramadhan and the summer vacation forced us to extend our intervention period to October. The principal author, a senior researcher of Pakistani origin, approached the women by mobilizing different channels such as Pakistani organizations and community centres to identify one informant in each of the four areas. After a time consuming process, including use of personal social circle and involvement of organization or community centres, including the Imams of the mosques in the study areas in the intervention areas, four key informants were recruited, and they further recruited participants through different channels such as personal phone calls, community gatherings and social media (Facebook). Women who did not show up for the first meeting despite having agreed to it, were contacted again to attend the second meeting through personal visits, phone calls and through women who attended the first meeting.

A Somali female research assistant was hired for the recruitment of the Somali women. She contacted women in her network in these four areas over the telephone, informed them about the intervention meeting and asked whether they were willing to participate. These women then provided contact information of other women in their network. The women who did not show up for the first meeting were visited by the research assistant at their homes to ask them to attend the next meeting.

As an incentive for participation we offered two lottery gift cards each worth NOK 500 after every meeting, in addition to serving of tea, coffee and refreshments etc. We offered an air ticket to Pakistan and Somalia for one woman from each of the respective groups drawn by lottery. The reason for this incentive was to motivate the participation of the target communities to the intervention.

#### Participants

Among the 915 Pakistani and 625 Somali women, who were living in these areas as indicated by The Statistics Norway (SSB), the key informants managed to invite a total of 401(42% of the Pakistani) and 485 (78% of Somali) women to one of the seven meetings arranged for each group. The Pakistani women who attended the meetings were 102 (11.5% of the total and 25% of invited), while the Somali women were 128 (14% of the total and 26% of invited).

The characteristics of the participants are presented in Table 1. This data was collected through the questionnaire distributed after the meeting and was answered by all the women who attended the meeting. The majority of the women from both countries attending the meetings were in the middle aged, married and had three or more children. The number of separated and divorced women was greater among Somali women. Pakistani women had secondary or higher education and the majority had taken a cervical cancer screening test at least once, while less than half of the Somali women had attended secondary or higher education, and only 45% had ever gone for a screening test.

Table 2 shows the questions asked by the participants grouped in three categories: cervical cancer, cervical cancer screening test and Human papilloma virus (HPV).

**Table 2:** Questions asked by the participants grouped in three categories. Questions raised by the participating women

Cervical Cancer Screening Test	
1. How to make the appointment for Pap test? 2. Why the duration of repeating Pap test is 3 years? What if something happens in between? 3. Why don’t GPs give us any information about the importance of cervical screening test? 4. Can we ask for a female doctor if we go for the screening test? 5. Can we take the test at our GPs office or we must travel to other places to get it done? 6. Can Pap test give the information about cancer of other parts of body like lung or breast? 7. Do we get any letters? (I haven’t received any letter from last 15 years).	
Cervical Cancer	
1. How can we diagnose cervical cancer? 2. What are the symptoms of cervical cancer in early stage and in later stage? 3. What is the likely age of getting cervical cancer? 4. Can cervical cancer be diagnosed during pregnancy? 5. What are the ways to prevent cervical cancer? If we have some cell changes, why the doctor tells us to wait for one year? Isn’t that dangerous? 6. What if we get the cancer in between? Why we must wait so long? Why don’t we get the treatment right away? 7. Can cell changes get back to normal? 8. Is urinary infection related to cervical cancer? 9. What is the incidence of cervical cancer among Pakistani women in Norway? 10. Can cervical cancer metastasize to other parts of the body like intestines, liver, etc.? 11. How can we get the tests for other organs of the body?	
HPV	
1. What is HPV? What are the symptoms of HPV infection? 2. If we get pregnant, then do we have the same symptoms of HPV or there will be any difference? 3. What is HPV vaccination? Is it necessary? What is the age limit for this vaccination? 4. Is there any research showing side effects of HPV vaccination? What are the symptoms of HPV? 5. Are unmarried women also at the risk of getting HPV infection and cancer? 6. What is HPV vaccination? Is it safe? Is it necessary? 7. Can you give us some information about the benefits and risks of HPV vaccination? 8. Should we give it our school going daughters? Is HPV vaccination recommended for unmarried women or those who have no sexual contacts? 9. Tell us about HPV in men? Causes and risks? 10. Can we get infected from men (if our husbands have)? Do men also need HPV vaccination?	

## Discussion

This intervention study was the first of its kind to be carried out among the two main immigrant groups in Norway. The intervention itself had a combination of elements that have been reported to be successful in other intervention studies carried out among immigrants in other parts of the world [23–25].

The intervention’s theoretical basis was Heron’s framework and the results of our earlier study using the ecological framework. Following the Heron’s theory, the intervention can be mainly seen as *prescriptive and informative,* as we tried to give the participants advice on the uptake of pap-smear, *and objective* information about the benefits of participating in the screening program and harms of non-participation and instructions on how to proceed. From the facilitative point of view, our intervention was mainly supportive and motivational, as we tried to answer questions and fears expressed by the women. As regards the *cathartic and catalytic elements*, we allowed the participants to express their experiences, anger, hesitations during the interactive meeting, *and* being judgemental in deciding whether to participate in the screening program or not. Through these elements we have tried in every possible way to encourage the women in participating in the screening program without being authoritative or confronting, but helping them in making a personal informed choice for participation considering both the harms of not participating and benefits of participation.

The first practical challenge was related to the high probability of contamination linked to the division of areas in randomization and control areas. The groups of migrants we chose have extensive internal networks that were broader than our defined areas. For this reason we had to extend the areas, and thus lost randomization even though we had explored previously the existence of mosques or immigrant shops in between. The lesson to learn from this is, although we were geographically able to identify immigrant majority areas but we should not forget the tight bonds that exist within the immigrant groups, which extend beyond the geographical boundaries.

The second challenge was related to the recruitment of women. This is in accordance with many other studies that have highlighted the methodological challenges of recruiting minority groups into research trials [26–29]. As previously reported by our group, the researcher should always try to develop a trust relationship with the participants, rather than just showing up at the door for recruitment purpose [15]. One strategy to overcome this fear is to have researchers from the same ethnic background to eliminate the element of mistrust and we adopted this strategy in our intervention. Also, we adopted community-based recruitment through immigrant organizations, mosques etc. in addition to recruitment through personal contacts. All these strategies have been reported as being helpful in trial settings [29]. Still, the attendance to the meetings was approximately one in four invited. Furthermore, the majority of young educated women had the opinion that a lot of this information can also be obtained from the internet, thus making it unnecessary to attend such meetings. However, among those who attended, we were able to ensure a highly diverse group regarding their level of education, age, and marital status and this information did attract the attention of older and uneducated women who are not familiar with digital technology and who probably have the lowest attendance to screening tests. Last, we encouraged diffusion of information whereby women who participated in the study will hopefully pass the information to others in their neighborhood.

The current intervention was a culturally adapted intervention in many respects, including targeting of language, gender, venue and development of material. Similar to our intervention study, other intervention have also been carried out among other immigrant groups [8–11, 23–25]. The majority of them were community based and included the use of community lay-health workers, linguistically-appropriate and culturally-tailored educational materials or navigation assistance to overcome the barriers to access the services. These interventions have resulted in increase in awareness and knowledge about cervical cancer with increase in screening participation [23]. Although we did not use community health workers for our intervention method, we did ensure that it was linguistically and culturally appropriate. Unlike other studies, we did not use written educational materials as women during the focus group discussion pointed out that sending of brochures, letters etc. would not be helpful. Rather, they wanted to have information given to them in seminar presentations by professionals. This strategy seems to have been correct in the light of our data, since approximately 30% among the women attending the meetings were unable to read any type of health related letters, brochures, posters etc. Similarly, the immigrant groups involved in this intervention came from oral and visual cultures, i.e. cultures that learn through listening and watching, and not through reading or writing. Inadequate health literacy among immigrant women has been reported by previous studies [30, 31].

Among the participating women, 25% had never taken cervical cancer screening test despite 81% of the women had children and it is a common practice to take a cervical test during antenatal visits. Several factors might explain this fact. The type of questions raised by the participants after the seminars points to a low level of awareness and knowledge regarding screening test among the women. This has also been reported by other studies [32]. Therefore, the information given by the researcher focused on cervical cancer in general, to raise the awareness and basic knowledge among the women. Lack of information given by the GPs to some immigrant groups has also been reported by our group (Møen, 2018 submitted). Additionally, as explained above, low health literacy and lack of knowledge about the health care system were widespread among the women. At the same time women had also expressed in the previous focus groups that they did not pay attention to the invitation letter sent by the cancer registry. We tried to address all these issues in our intervention. Once women got some information, the participants wanted to know more about the disease and its causes and how it could be prevented. After the presentation women seemed to realize the importance and sensitivity of the issue, as shown by the data that 78% of Pakistani whereas 90% of the Somali women replied that they will contact the GP for the test after attending this meeting.

Majority of the women who did not agree to take a pap smear were those who had already undergone screening either in the previous year (26% Pakistani & 15% Somali) or 2 years ago (46% Pakistani & 33% Somali). However, most Somali and Pakistani immigrants in Norway have challenges in coping with the new way of life in their host country. Solving their day-to-day problems in life may divert their attention away from participation in health programs and research and intention to take the test does not necessarily change behavior.

In order to see the effect of our intervention we plan to measure the effect of the intervention between the intervention and control areas by analysing the data from the national cancer register which records personal information on cervical cancer screening.

## Conclusions

We have developed and implemented an intervention among immigrant women to increase their participation in cervical cancer screening following the existing recommendations for culturally adapted studies. Still, we confronted methodological and recruitment challenges that are described in this paper for the future advancement of the field. Although we had initially designed it to be a randomized controlled trial, we had to restrict ourselves to a community-based intervention with a non-randomised control group to avoid contamination. This is to say, we had to give up randomisation which is considered to be an important component of intervention studies.

Behavioural interventions are complex and when targeting them to immigrants adds further complexity [33]. However, it is of fundamental importance to recognise the specificity of promoting health interventions within immigrant populations. Although, such intervention methods are more time consuming, need extensive resources and personal commitment, we hope to demonstrate in the near future that the effectiveness of interventions such as ours provides basis for the justification and commitment of resources to this approach in health promotion research. We further hope that this intervention will allow future researchers to learn from our experience and challenges and eventually develop more targeted interventions for immigrant groups.

## Additional files

Additional file 1: Questionnaire. (PDF 445 kb)

Additional file 2: Power Point presentation. (PDF 2210 kb)

## Data Availability

The dataset used and analysed during the current study are available from the corresponding author on request.
