# Supplementary material for: A community-based intervention to increase participation in cervical cancer screening among immigrants in Norway
Source: BMC Med Res Methodol. 2019 Jul 12;19:147. doi: 10.1186/s12874-019-0795-8 (PMC6626362; doi:10.1186/s12874-019-0795-8)
Supplement: Supplementary file 1 — Questionnaire. (PDF 445 kb) [file 12874_2019_795_MOESM1_ESM.pdf]

Dear Madam,

Thank you for taking part in the meeting and completing this questionnaire!

We want to know the effect of these meetings to increase attendance to cervical cancer screening. Please ask the organizers if there is any question that you do not understand and give the completed questionnaire back to the organizers before you leave the meeting.

By answering this questionnaire, you accept that we use this information only for the purpose explained to you. All information will be treated in strict confidence.

محترمہ ! اس تحقیقی مطالعے اور میٹنگ میں حصہ لینے کیلئے آپ کا شکریہ۔ ہم امید کرتے ہیں کہ آپ ٹھیک ٹھاک ہیں۔
اس سوال نامہ کے ذریعہ ہم اس میٹنگ کے سرویکل کینسراسکرینیگ میں شرکت پر اثرات جاننا چاہتے ہیں۔
اگر کوئی سوال ہو تو آپ منتظمین سے پوچھ سکتی ہیں۔
جانے سے پہلے مکمل شدہ سوال نامہ ضرور واپس کریں۔ اس سوالنامہ کو پُر کرکے آپ همیں ان معلومات کوتحقیقی مطالعے کے لیئے استعمال کرنے کی اجازت دے رہی هیں۔
ہم آپ کو یقین دلاتے ہیں کہ یہ تمام معلومات صرف تحقیقی مطالعے کے لیئے استعمال ہو گی۔
سوالیہ فارم مکمل کرنے کیلئے آپ کا شکریہ۔ اس میں 15 منٹ سے زیادہ نہیں لگیں گے۔ درست جواب پر کراس (x) لگائیں۔

1. How old are you, in years?

Under 25 ❒ 25 ❒ 26 - 45 ❒ 46 - 69 ❒ 70 or older ❒

1- آپ کی عمر؟

25 ❒ یا اُس سے کم 25 ❒ 26 - 45 ❒ 46 – 69 ❒ 70 ❒ یا زیادہ

2. Do you have children?

Yes ❒ No ❒

2- آپ کے بچے ہیں؟

ہاں ❒ نہیں ❒

3. If you have children, how many do you have?

1 ❒ 2 ❒ 3 ❒ 4 ❒ 5 or more ❒

3- اگر ہیں تو کتنے ہیں؟

1 ❒ 2 ❒ 3 ❒ 4 ❒ 5 یا زیادہ ❒

4. What is you marital status?

Single ❒ Separated ❒ Married ❒ Divorced ❒ Widowed ❒ Other ❒

4- آپ کی ازدواجی حیثیت کیا ہے؟

غیر شادی شدہ ❒ علیحدگی ❒ شادی شدہ ❒ طلاق یافتہ ❒ بیوہ ❒ اور کوئی ❒

5. Which country were you born in?

Norway ❒ Somalia ❒ Pakistan ❒ Other ❒

5- آپ کہاں پیدا ہوئیں ہیں؟

ناروے ❒ پاکستان ❒ کہیں اور ❒

6. Which language do you speak most often at home? (*You can put two X if you speak more than one*)

Norwegian ❒ Urdu ❒ Somalian ❒ English ❒ Other ❒

6- آپ گھر میں عمومًا کون سی زبان بولتی ہیں؟(ایک سے ذیادہ جواب دے سکتے ہیں۔)

❐ نارویجن ❐ اُردو ❐ پنجابی ❐ انگلش ❐ کوئی اور

7. How many years of education have you completed altogether?

No school ❐ Primary school ❐ Secondary school ❐ Higher education ❐

7- آپ نے کتنی تعلیم حاصل کی ہے؟

❐ بالکل نہیں ❐ پرائمری ❐ سیکنڈ ری ❐ یونیورسٹی

8. Do you know how to make an appointment with your “fastlege”?

Yes ❐ No ❐

8- کیا آپ جانتی ہیں کہ ڈاکٹر سے ملاقات کا وقت کیسے لینا ہے؟

❐ ہاں ❐ نہیں

9. Have you ever taken a Pap smear?

Yes ❐ No ❐

9- کیا آپ نے کبھی 'پیپ ٹیسٹ' کرایا ہے؟

❐ نہیں ❐ ہاں

10. If you have taken a Pap smear, how long is it since the last time you did so?

1 year ❐ 2 years ❐ 3 years ❐ 4 years or more ❐

10- اگر آپ نے 'پیپ ٹیسٹ' کرایا ہے، تو اس کو تقریباً کتنا عرصہ ہوا ہے؟

❐ 4 سال یا زیادہ ❐ 3 سال ❐ 2 سال ❐ 1 سال

11. After this meeting, do you think you should contact your fastlege to take a Pap smear?

Yes ❐ No, because I have already done ❐ No, it is not necessary ❐

11- کیا آج کی میٹینگ کےبعد آپ اپنے ڈاکٹر سے 'پیپ ٹیسٹ' کے لئے رابطہ کریں گی؟

❐ ہاں ❐ نہیں، کیونکہ میں یہ پہلے کرا چُکی ہوں ❐ نہیں، یہ غیر ضروری ہے

12. How often do you need someone to help you when you read instructions, pamphlets, or other written material from your doctor or pharmacy?

Never ❐ Rarely ❐ Sometimes ❐ Often ❐ Always ❐

12- جب آپ کوکوئی تحریر مثلاً کتابچہ ، ہدایات وغیرہ ڈاکٹر یا ادویات کی دوکان سے ملے تواُس کو پڑھنے کے لئے آپ کو کسی کی مدد درکار ہوتی ہے؟

❐ بالکل نہیں ❐ شاذونادر ❐ کبھی کبھار ❐ اکثر ❐ ہمیشہ

Thank you very much for coming. Please make an appointment with your “fastlege” to take a cervical cancer screening and invite your friends to do the same. It might save your life!

آپ کی شرکت کا شکریہ۔ آج ہی اپنے ڈاکٹر سے پیپ ٹیست کیلئے رابطہ کریں اور دوستوں کو بھی مشورہ دیں۔ یہ آپ کی جان بچا سکتا ہے۔

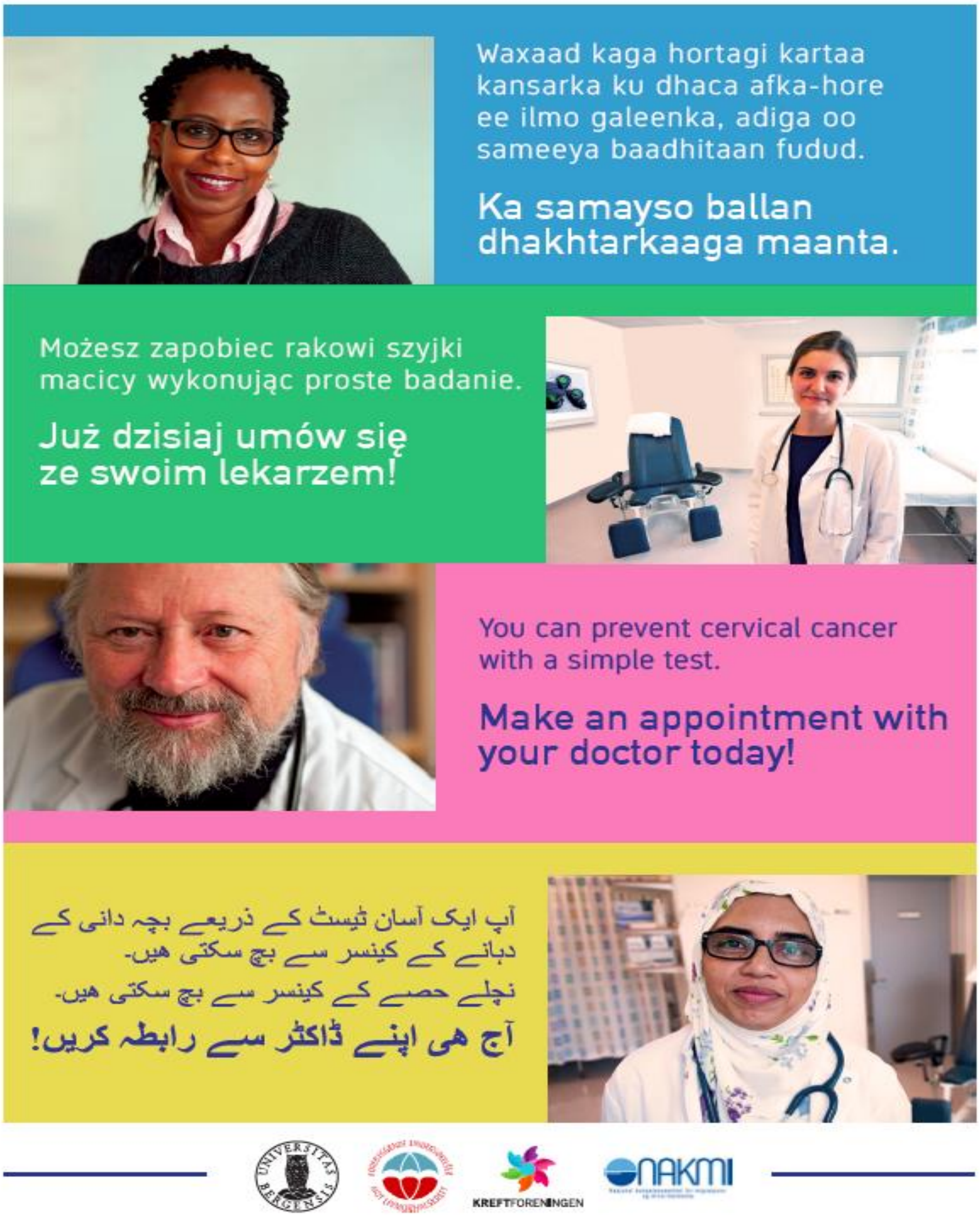
Waxaad kaga hortagi kartaa kansarka ku dhaca afka-hore ee ilmo galeenka, adiga oo sameeya baadhitaan fudud.
Ka samayso ballan dhakhtarkaaga maanta.
Możesz zapobiec rakowi szyjki macicy wykonując proste badanie.
Już dzisiaj umów się ze swoim lekarzem!
You can prevent cervical cancer with a simple test.
Make an appointment with your doctor today!
آپ ایک آسان ٹیسٹ کے ذریعے بچہ دانی کے دہانے کے کینسر سے بچ سکتی ھیں۔
نچلے حصے کے کینسر سے بچ سکتی ھیں۔
آج ھی اپنے ڈاکٹر سے رابطہ کریں!
KREFTFORENINGEN

Write your contact information at the back of this page if you want to take part in the lottery!

اگر آپ لاٹری میں حصہ لینا چاہتی ہیں تو اپنا نام اور فون نمبر اس صفحہ کے پیچھے لکھیں۔

This information is not for research, only to contact you if you win the lottery prize.

یہ معلومات صرف انعام کیلئے ہیں۔ تحقیق کیلئے نہں!

Name **نام** ____________________________

Telephone number **ٹیلیفون نمبر** _________________________
