# Supplementary material for: A community-based intervention to increase participation in cervical cancer screening among immigrants in Norway
Source: BMC Med Res Methodol. 2019 Jul 12;19:147. doi: 10.1186/s12874-019-0795-8 (PMC6626362; doi:10.1186/s12874-019-0795-8)
Supplement: Supplementary file 2 — Power Point presentation. (PDF 2210 kb) [file 12874_2019_795_MOESM2_ESM.pdf]

NAKMI.NO
Kompetanse – Helse – Migrasjon

Cervical Cancer Intervention Meeting

UNIVERSITAS BERGENSIS

Samera Azeem Qureshi MD, PhD
Prabhjot Kour MD, MPhil

NORWEGIAN **CANCER** SOCIETY

OUR CERVICAL CANCER PROJECT
سرویکل کینسر پروجیکٹ

Goals **مقاصد**

- To evaluate the effectiveness of two different community-based strategies to increase the attendance to the cervical cancer screening prevention program.

سرویکل کینسر اسکرینگ پروگرام میں شرکت کو بڑھانے کے لیئے دو مختلف طریقوں کے اثرات کو جانچنا۔

- Effect of the interventions measured quantitatively will be compared to each other

پھر ان دونوں مختلف طریقوں کا آپس میں موازنہ کرنا ہے۔

CERVICAL CANCER PROJECT
سرویکل کینسر پروجیکٹ

Bergen:

Target Group: ٹارگٹ گروپ

- GPs
- Nurses
- Midwives

Method: طریقہ کار

- Posters
- Post- cards
- Meetings

Oslo:

Target Group: ٹارگٹ گروپ
Pakistani and Somali women
پاکستانی اور صومالی خواتین

Place: جگہ
Lørenskog
Bærum
Asker
Drammen

Method: طریقہ کار

Information meetings معلوماتی میٹنگ

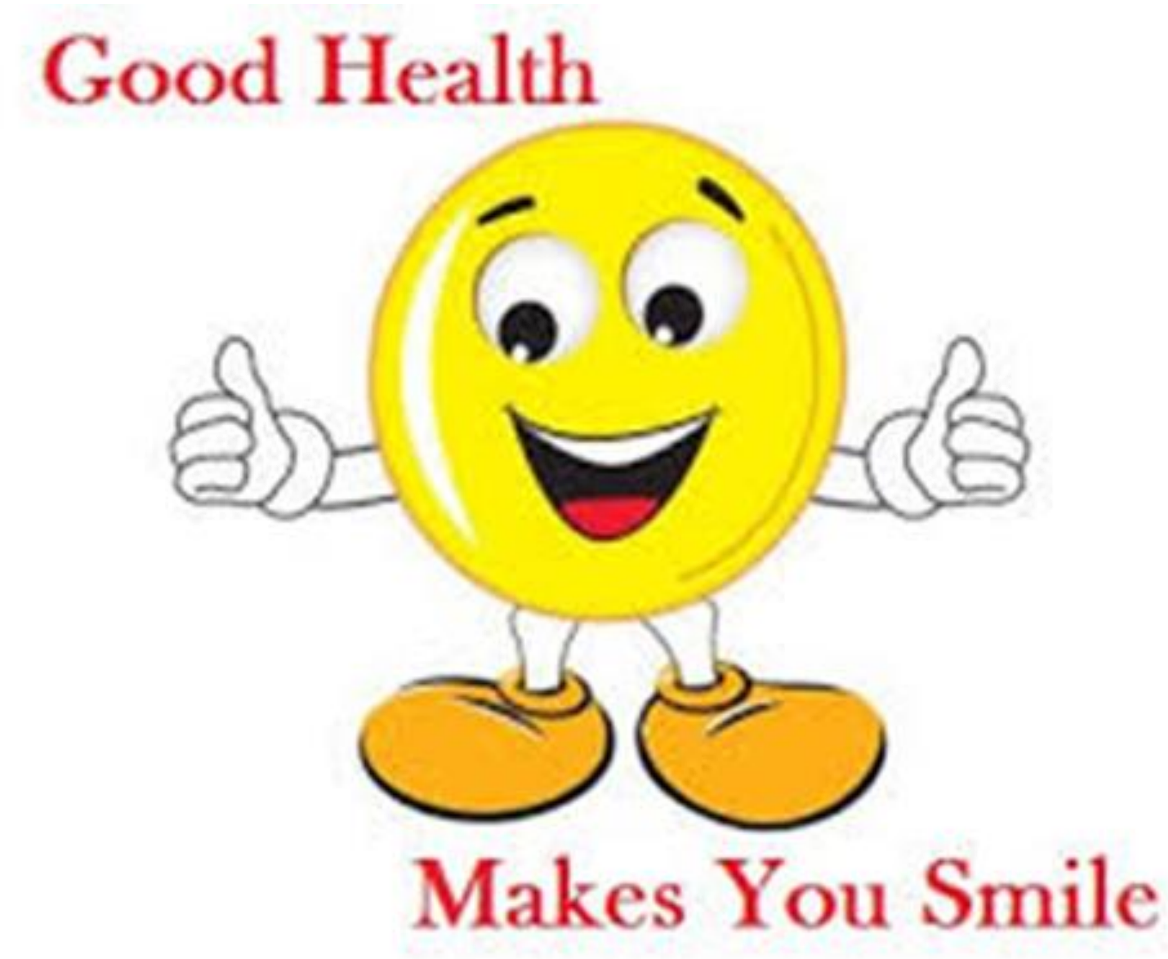
Good Health
Makes You Smile

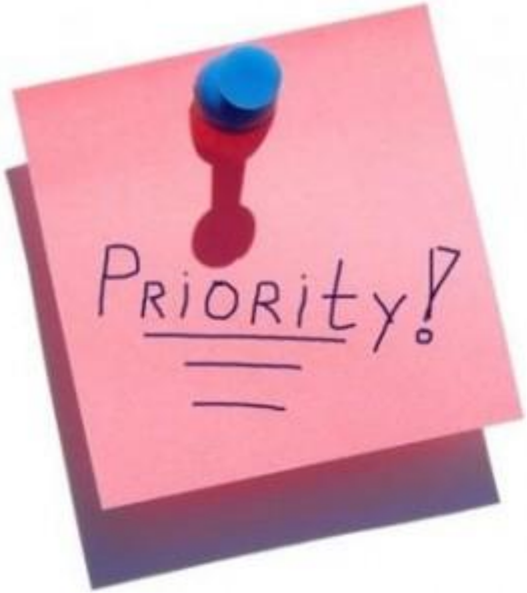

Health

صحت

Take good care of your Body

اپنے جسم کا بہتر طریقے سے خیال رکھنا۔

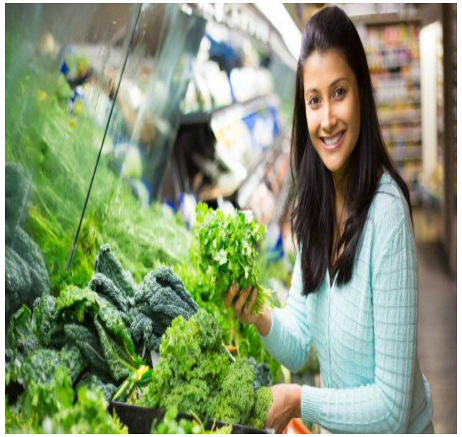

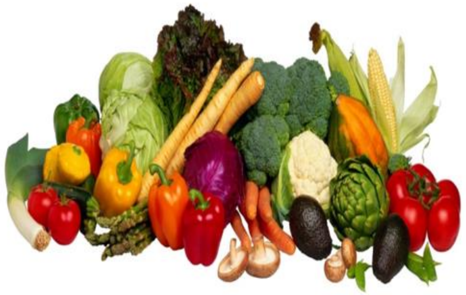

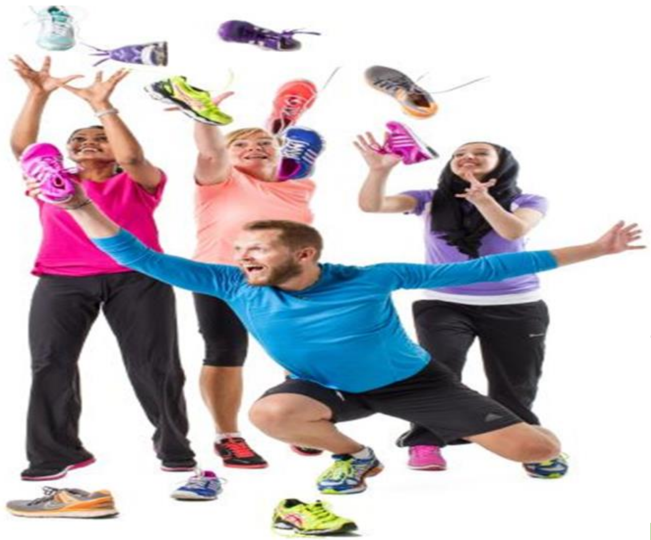

Healthy Lifestyle

Eat Healthy

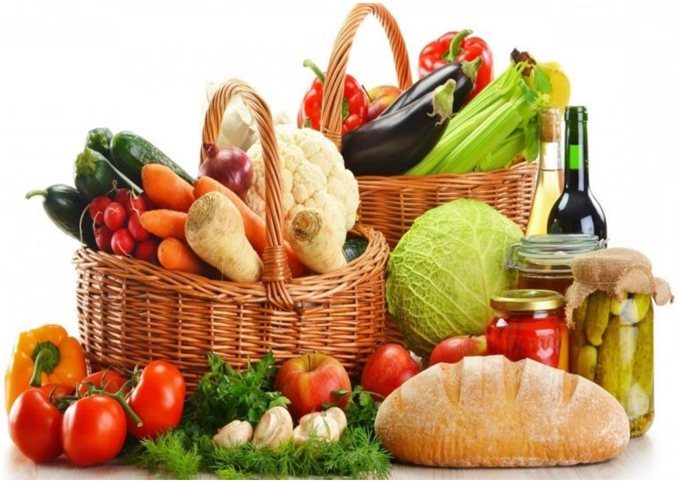

Be Active

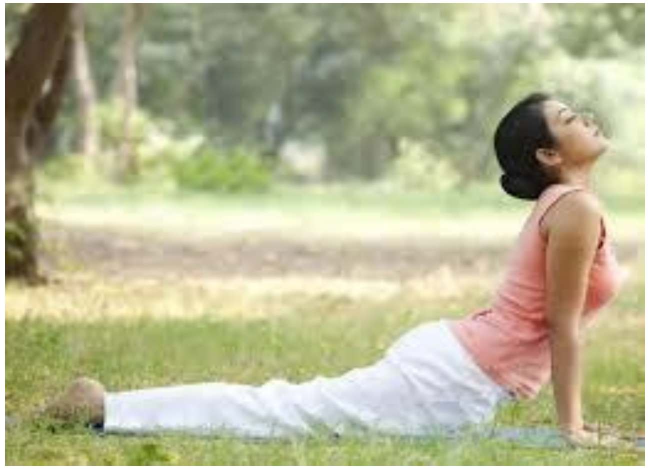

Drink Water

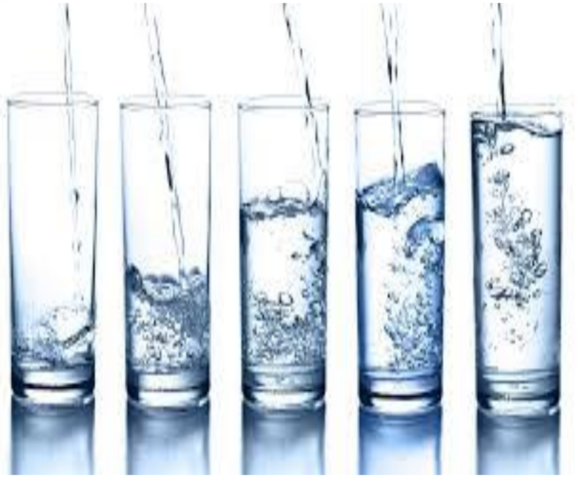

Enough sleep

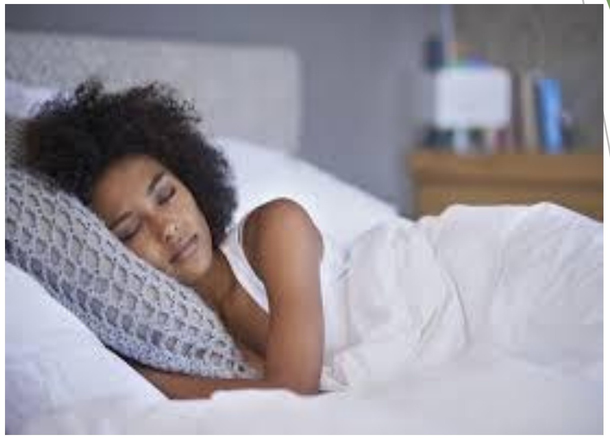

Regular Check-up

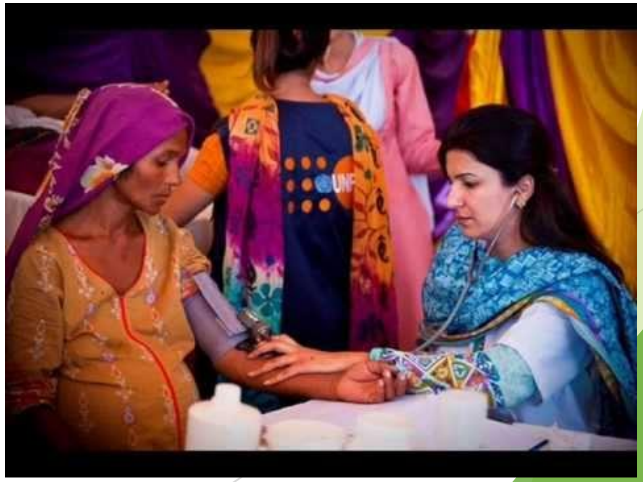

Cancer
کینسر

Frequently asked questions about cancer?

کینسر کے بارے میں عمومی سوالات؟

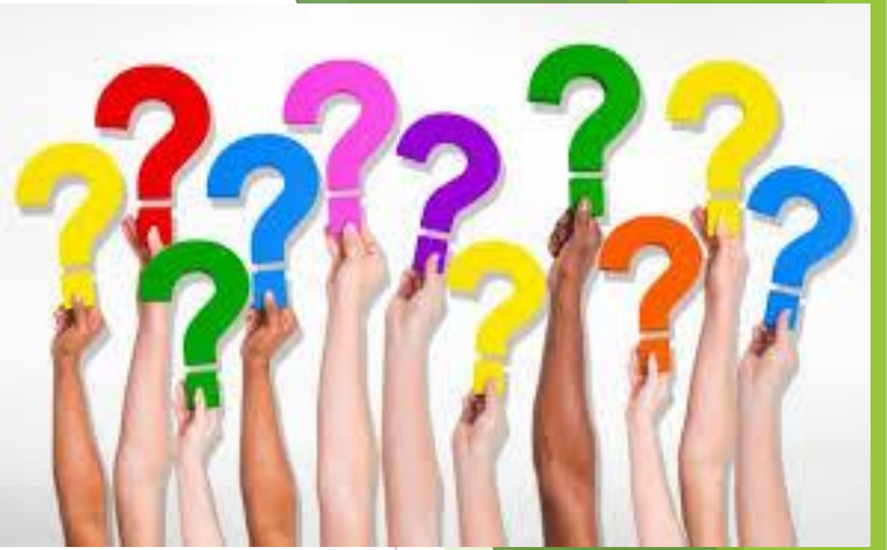

Can I have cancer without knowing?

کیا مجھے جانے بغیر کینسر ہو سکتا ہے؟

Will I have to take medicines all my life?

کیا مجھے اب ساری عمر دوائیاں استعمال کرنی ہوں گی؟

Is cancer dangerous?

کیا کینسرخطرناک ہوتا ہے؟

Will I be healthy again?

کیا میں تندرست ہو سکتی ہوں؟

Is it contagious?

کیا کینسر چھوت کی بیماری ہے ؟

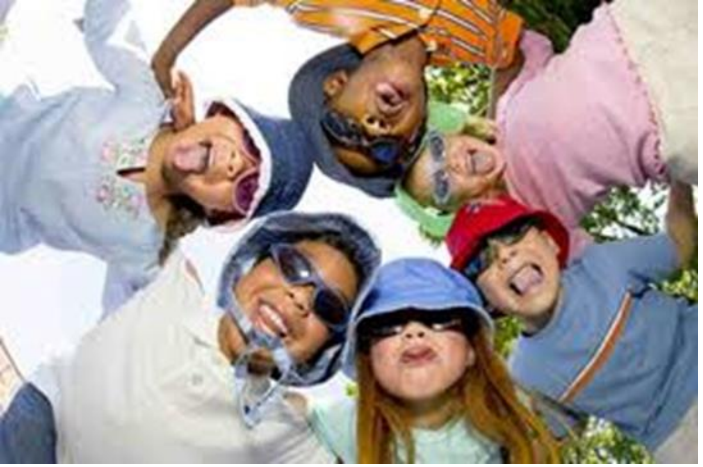

WHAT IS CERVICAL CANCER?

بچہ دانی کے نچلے حصے کا کینسر کیا ہے؟

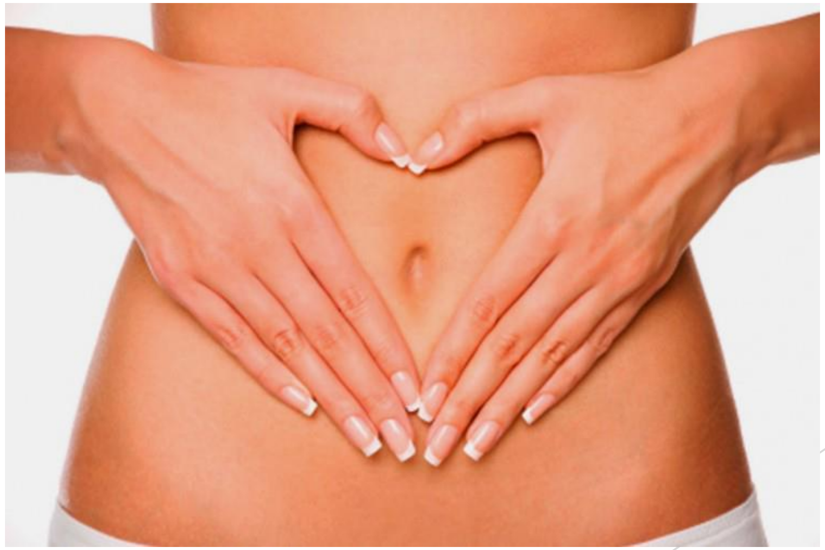

WHAT IS CERVICAL CANCER?

- Cancer that starts in the CERVIX- the lower part of the uterus that connects to the vagina

بچہ دانی (رحم) کے نچلے حصے کے کینسر
کو (سرویکل کینسر) کہتے ہیں۔

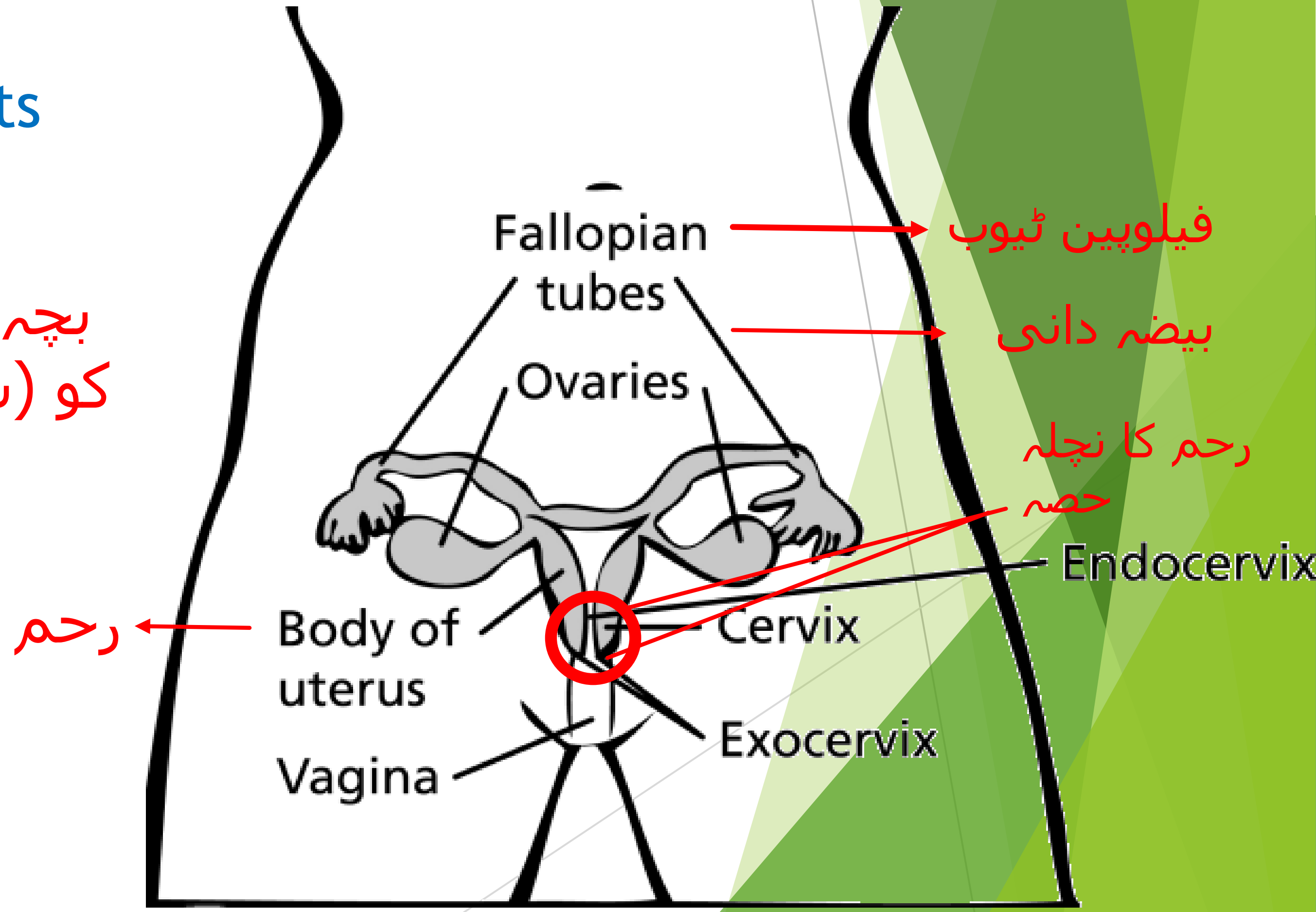

WHAT IS CERVICAL CANCER?

- Cancer starts when cells in the body begin to grow out of control.

جسم میں خلیات کے کنٹرول سے باہر اضافہ کو کینسر کہتے ہیں۔ اور یہ جسم کے کسی حصے میں شروع ہو سکتا ہے۔

- Cells in nearly any part of the body can become cancer, and can spread to other areas of the body.

جسم کے تقریبا کسی بھی حصے کے خلیات کینسر بن سکتے ہیں، اور یہ جسم کے دیگر حصوں تک پھیل سکتا ہے۔

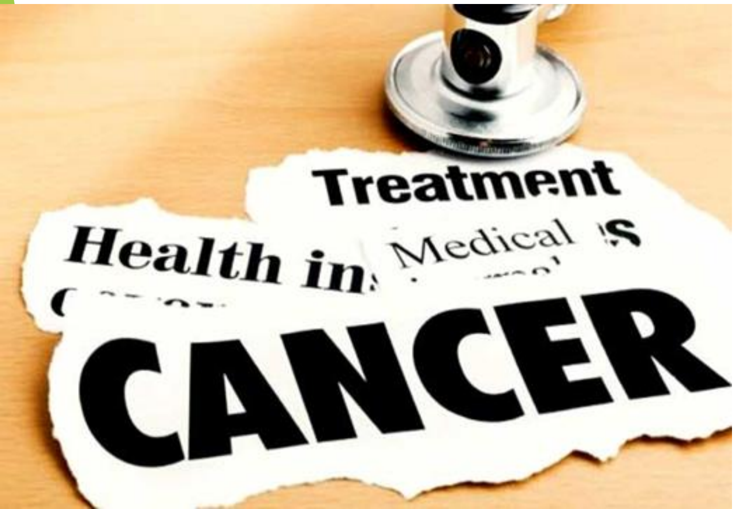

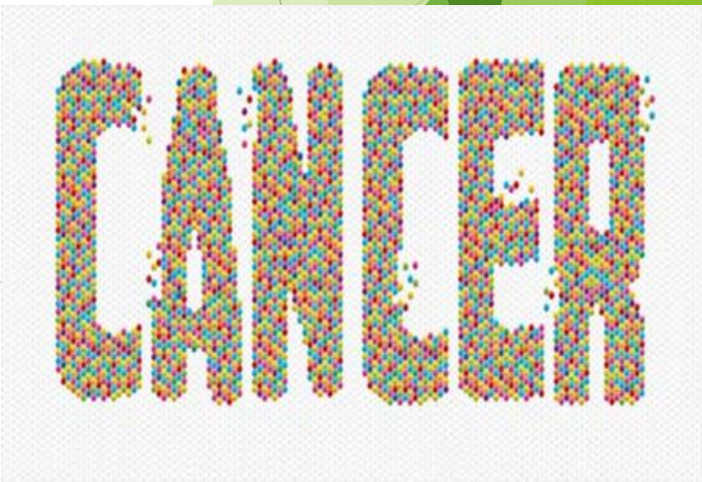

WHAT IS CERVICAL CANCER?

- Most cervical cancers start in the cells lining the cervix.

زیادہ تر کینسر رحم کے نچلے حصے کے خلیوں میں شروع ہوتے ہیں۔

- These cells do not suddenly change into cancer. Instead, the normal cells of the cervix first gradually develop pre-cancerous changes that may turn into cancer.

یہ خلیات اچانک کینسر نہیں بنتے، بلکہ ان میں بتدریج تبدیلی آتی ہے جو کے بعد میں کینسر بن جاتی ہے۔

- These changes can be detected by the PAP TEST and treated to prevent cancer from developing.

یہ تبدیلیاں **پیپ ٹیسٹ** کے ذریعے پتہ چلائی جا سکتی ہیں اور کینسر سے بچا جا سکتا ہے۔

Causes of Cervical Cancer
کینسر کی وجوہات

The cause of nearly all cervical cancer is human papilloma virus or HPV.

تقریباً تمام سرویکل کینسر کی وجہ ہیومن پیپیلوما وائرس(ایچ پی وی) ہے۔

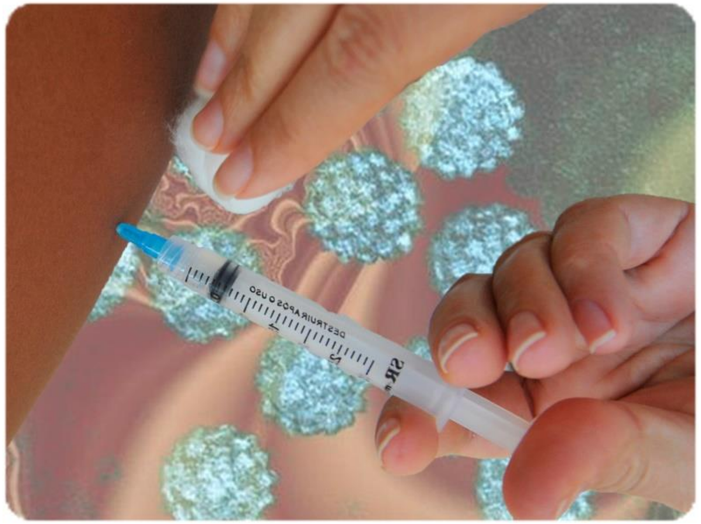

Risk factors for cervical cancer
کینسر کے عناصر

- HPV infection ایچ پی وی انفیکشن
  - Extremely common in women who have ever had sex

    یہ انفیکشن ہر اس خاتون کو ہو سکتا ہے جس نے جنسی ملاپ کیا ہو۔
  - Having an HPV infection does NOT mean you WILL get cervical cancer!

    ایچ پی وی انفیکشن کا مطلب یہ نہیں کے آپ کو کینسر ہو سکتا ہے۔
    - In most cases, HPV infection will clear on its own.

      ذیادہ تر یہ خود ہی ٹھیک ہو جاتا ہے۔
    - Only women with persistent HPV (where the virus does not go away) are at risk for cervical cancer.

      صرف وہ خواتین جن کو مسلسل انفیکشن کی شکایت رہتی ہو کو خطرہ ذیادہ ہے۔

Risk factors for cervical cancer
کینسر کے عناصر

- **Not getting screened- is the biggest risk factor.**

اسکرین نہ کروانا۔ سب سے بڑاعنصر ہے۔

- Immunosuppressed

قوت مدافیعت کا کم ہونا مثلاً

- HIV
- Diabetic
- Other

Cervical cancer screening
سروائیکل کینسر اسکریننگ

- Screening is testing to find cancer, or other diseases, early in people who have no symptoms.

اسکریننگ سے کینسر اور دوسری بیماریوں کا پتہ چلایا جا سکتا ہے۔

- Screening can help find cancers when they are small and have not spread – when they have a better chance of being cured.

اسکریننگ سے کینسر کو پھیلنے سے پہلے روکا جا سکتا ہے۔ جب کے علاج ممکن ہوتا ہے۔

Cervical cancer screening
سرویکل کینسر اسکرینیگ

Cervical cancer screening is done with

سرویکل کینسر اسکرینیگ دو طریقوں سے ہو سکتی ہے۔

- Pap Test

پیپ ٹیسٹ

- HPV test

ایچ پی وی ٹیسٹ

Cervical cancer screening

سرویکل کینسر اسکرینیگ

- What is a Pap test?

پیپ ٹیسٹ کیا ہے؟

- A test which collects cells from the surface of the cervix to check for any abnormal cells

اس ٹیسٹ کے ذریعے سروکس کے خلیوں کو اکٹھا کر کے ان کا معائنہ کیا جاتا ہے۔ تاکہ بیمار خلیوں کا پتہ چلایا جا سکے۔

Cervical cancer screening

سرویکل کینسر اسکرینیگ

- Abnormal cells can be removed or treated before cervical cancer develops.

بیمار خلیوں کا کینسر بننے سے پہلے علاج ممکن ہے۔

- When cancer is detected early, it is easier to treat.

کینسر کی جلد تشخیص علاج کو ممکن کر دیتی ہے۔

- A pelvic exam is NOT a Pap test; ONLY a Pap test can find early cervical cancer or pre- cancer.

نچلے حصے کا معائنہ پیپ ٹیسٹ نہیں ہوتا۔ صرف پیپ ٹیسٹ کے ذریعے کینسر کا پتہ چلایا جا سکتا ہے۔

Pap Test
پیپ ٹیسٹ

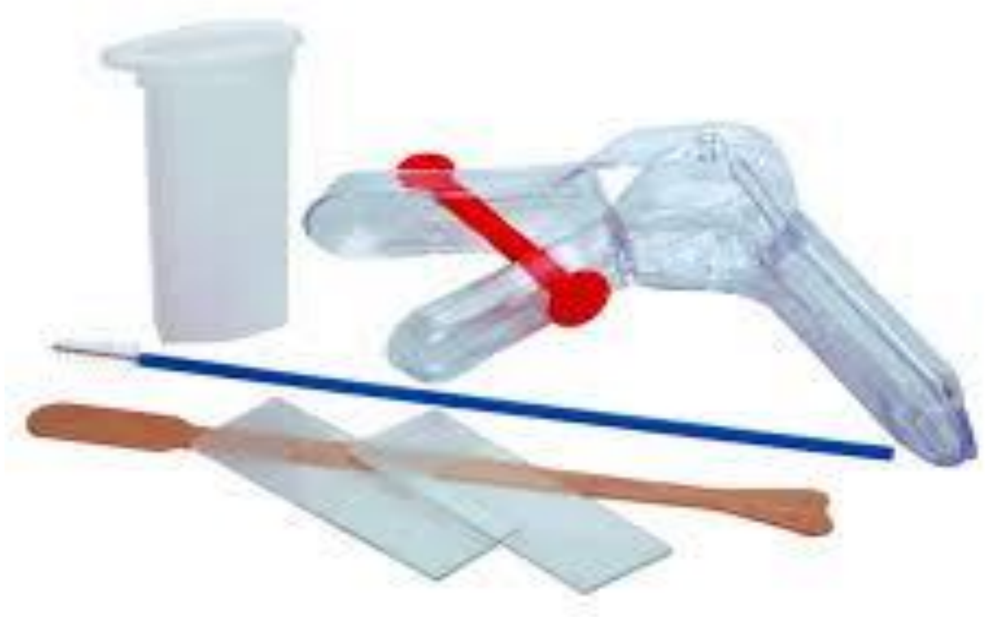

Instruments for pap test
پیپ ٹیسٹ کے اوزار

Pap Test Video

پیپ ٹیسٹ

Video

Pap Test
پیپ ٹیسٹ

- Pap tests are very accurate and regular

پیپ ٹیسٹ بالکل صحیح رزلٹ دیتا ہے۔

- Pap screenings reduce cervical cancer rates and mortality by 80%.

پیپ ٹیسٹ سروہکل کینسر اور اس کی وجہ سے اموات کو اسی فیصد تک کم کر دیتا ہے۔

Norwegian screening Guidelines
نارویجن اسکرینیگ گائیڈ لائینز

- All women between 25- 69 years every 3 years

25 -69 سال کی تمام خواتین کو ہر تین سال بعد ٹیسٹ کروانا چاہیئے۔

- The Cancer Registry, which runs NCCSP, sends letters to women between 25 and 69 years old who have a test taken less often than every third year

کینسر رجسٹری جو کہ نیشنل سرویکل کینسر اسکرینگ پروگرام چلاتا ہے۔ اس عمر کی تمام خواتین جو کے تین سال بعد بھی ٹیسٹ نہیں کرواتی کو یاد دہانی کے خطوط ارسال کرتا ہے۔

Norwegian screening Guidelines
نارویجن اسکرینیگ گائیڈ لائینز

How to take the test
ٹیسٹ کے لیئے ڈاکٹر سے رابطے کا طریقہ

- You can make an appointment at your General practitioner (GP).
  اپنے Fastlege سے ٹائم لیں۔
- The doctor does a gynaecological examination and takes samples from the cervix.
  وہ آپ کے نچلے حصے کے معائنہ کے بعد Sample لے گی۔

Payment or fee
فیس کی ادائیگی

- General practitioners NOK- 136
  Fastlege کی فیس NOK 136
- Test equipment- NOK 54
  اوزار NOK 54
- Ask what the fee is when you make an appointment.
  ڈاکٹر سے رابطہ کے وقت فیس کا بھی پوچھ لیں۔

Make an Appointment Today!

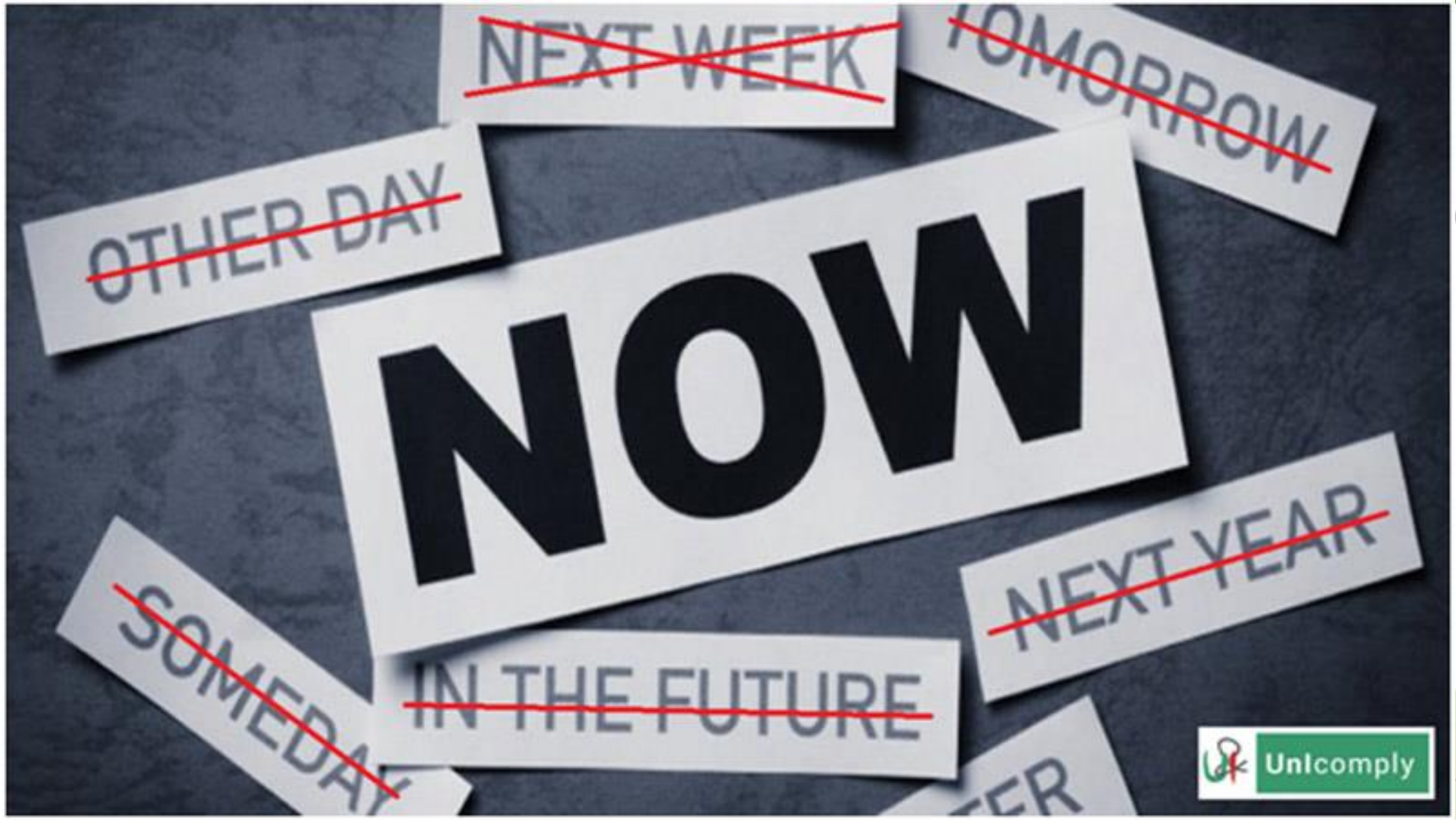

ابھی اپنے ڈاکٹر سے رابطہ کریں!

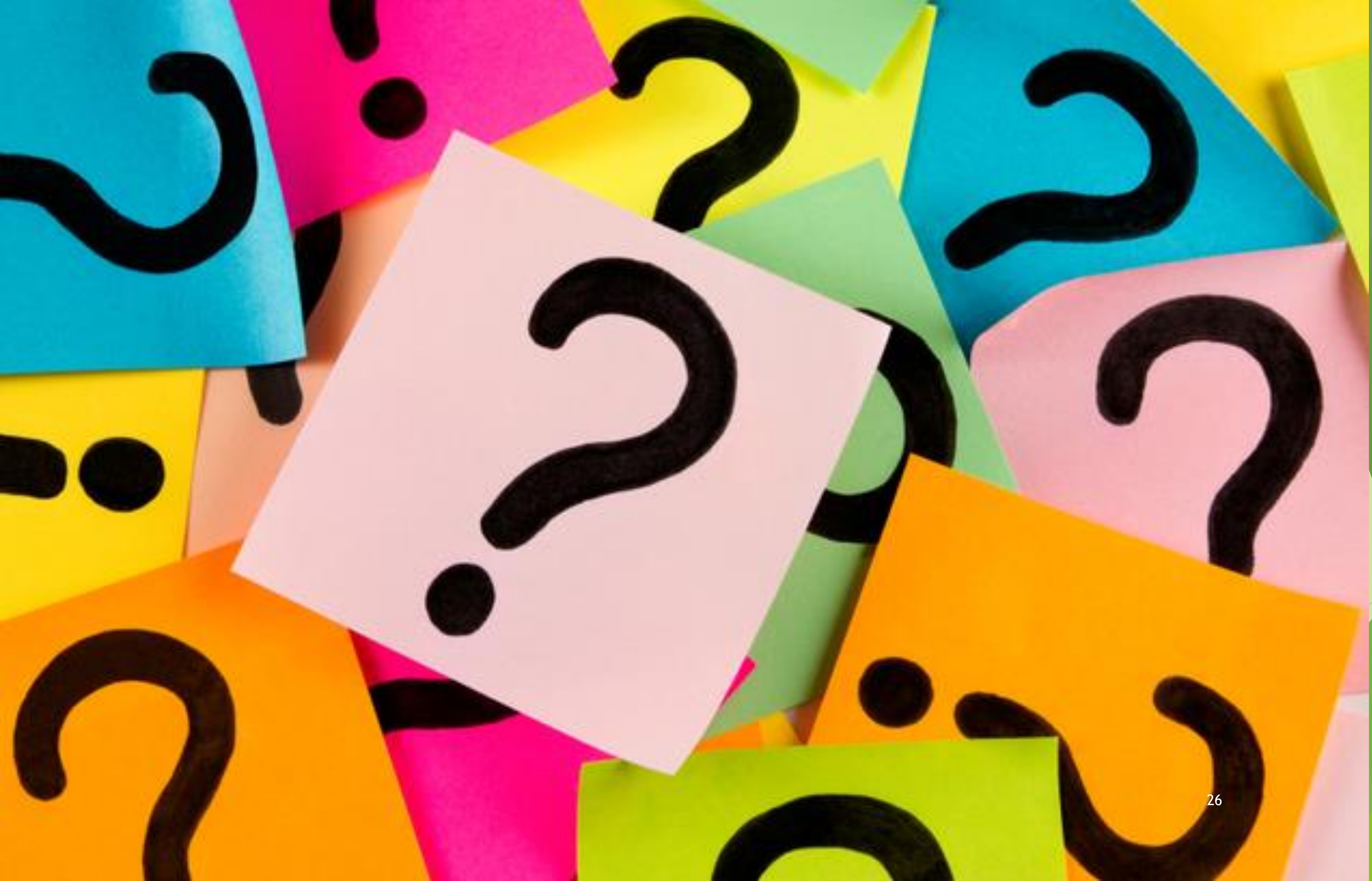

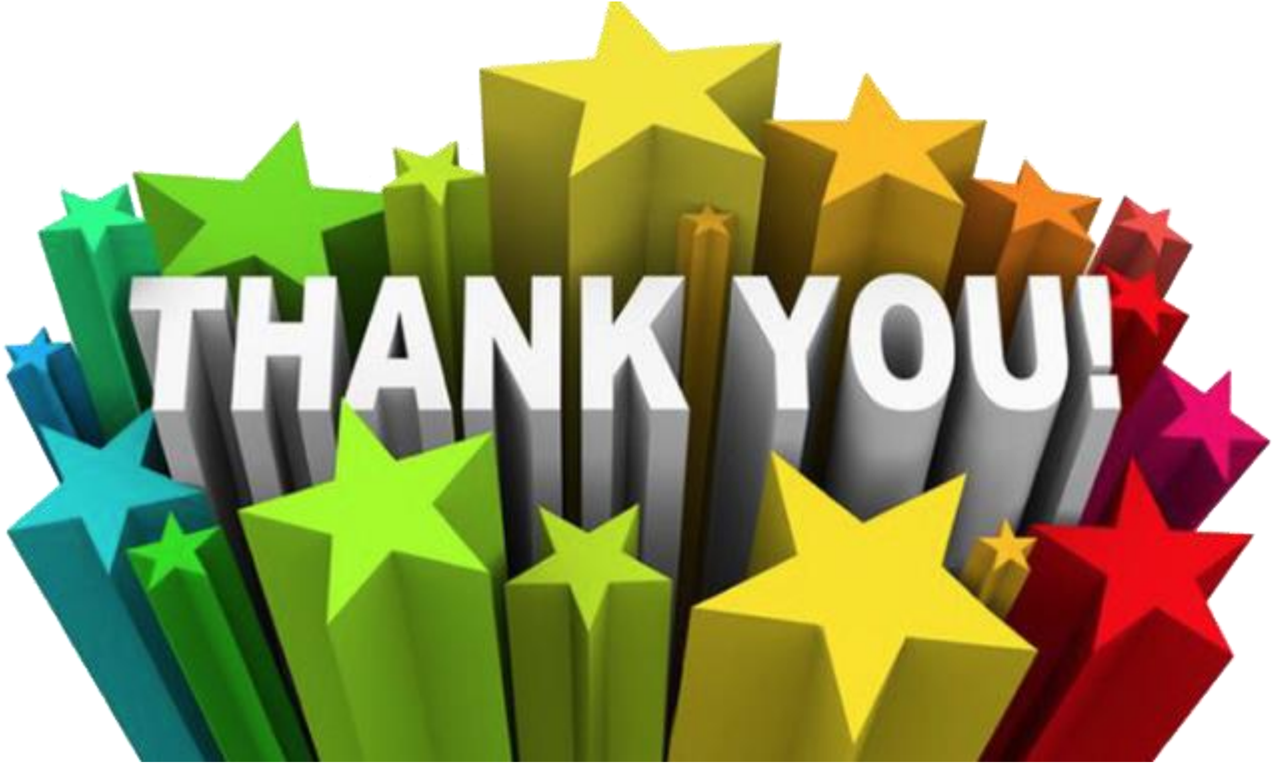
THANK YOU!

HPV vaccines

- Safe and effective
- Recommended for all girls and boys ages 11-12
- Requires 3 injections
- **Protect against cervical and many other cancers linked to HPV infection**
- Routine cervical cancer screening is still necessary for women who have been vaccinated

Why don’t parents get their kids vaccinated against HPV?

- Seen as not needed/necessary
- Safety concerns
- They don’t know about the HPV vaccines
- Not recommended by the doctor
